# Radiocarbon evidence of river transport and food web uptake of old carbon in Lake Athabasca, Canada

**DOI:** 10.1038/s41598-025-15926-5

**Published:** 2025-08-26

**Authors:** John Chételat, Craig Hebert, Christine McClelland, Sarah Greenwood

**Affiliations:** Environment and Climate Change Canada, National Wildlife Research Centre, Ottawa, ON Canada

**Keywords:** Ecosystem ecology, Carbon cycle

## Abstract

Rivers are major conduits that connect terrestrial carbon cycling with downstream receiving environments. In large lakes, the utilization of river-transported carbon in biological production remains poorly constrained. Lake Athabasca, the eighth largest lake in Canada, receives inflow from the Athabasca River and a large boreal wetland, the Peace-Athabasca Delta. Radiocarbon (^14^C) was measured to trace the transport of old carbon into western Lake Athabasca and its influence on a modern lake food web by distinguishing the ages of river and lake carbon pools. The fraction of modern carbon in surface sediments showed river inputs of old organic carbon extended at least 50 km into the lake. Old carbon was incorporated into the food web via surface water plankton, and it subsidized ~ 20–50% of carbon in vertebrate consumers, specifically fish and aquatic-feeding birds. These measurements of old carbon showed extensive river influence in western Lake Athabasca (likely > 1500 km^2^) and high connectivity of river loads with biological uptake.

**Supplementary Information:**

The online version contains supplementary material available at 10.1038/s41598-025-15926-5.

## Introduction

Radiocarbon is a powerful isotopic tracer that can be used to evaluate the mixing of water masses by revealing carbon ages and source(s)^1,2^. The radioactive isotope of carbon (^14^C) has a half-life of 5700 years, and environmental reservoirs such as soils, groundwater, surface water and the atmosphere vary in the age of the ^14^C pool. Thus, environmental gradients of radiocarbon provide a means to trace physical mixing processes^3,4^. Radiocarbon can also be applied to study biological processes including the identification of carbon sources supporting food webs^2,5^. Present-day aquatic animals are typically composed of modern carbon because biomass and energy ultimately originate from photosynthetic fixation of atmospheric carbon dioxide. However, pools of old carbon such as from terrestrial soils and peatlands, weathering of carbonate rocks, melting glaciers and thawing permafrost are biologically labile and can be incorporated into biomass in modern food webs^6–8^. While food web incorporation of old carbon has been reported for small aquatic ecosystems, estimates of river subsidies of old carbon to large lakes remain poorly constrained. An exception is the Great Lakes where there are limited sources of old carbon and modern carbon has been shown to support biological production^9,10^. Additional research is needed to further refine and characterize the role of old carbon in the carbon cycle^11,12^.

**Fig. 1:**
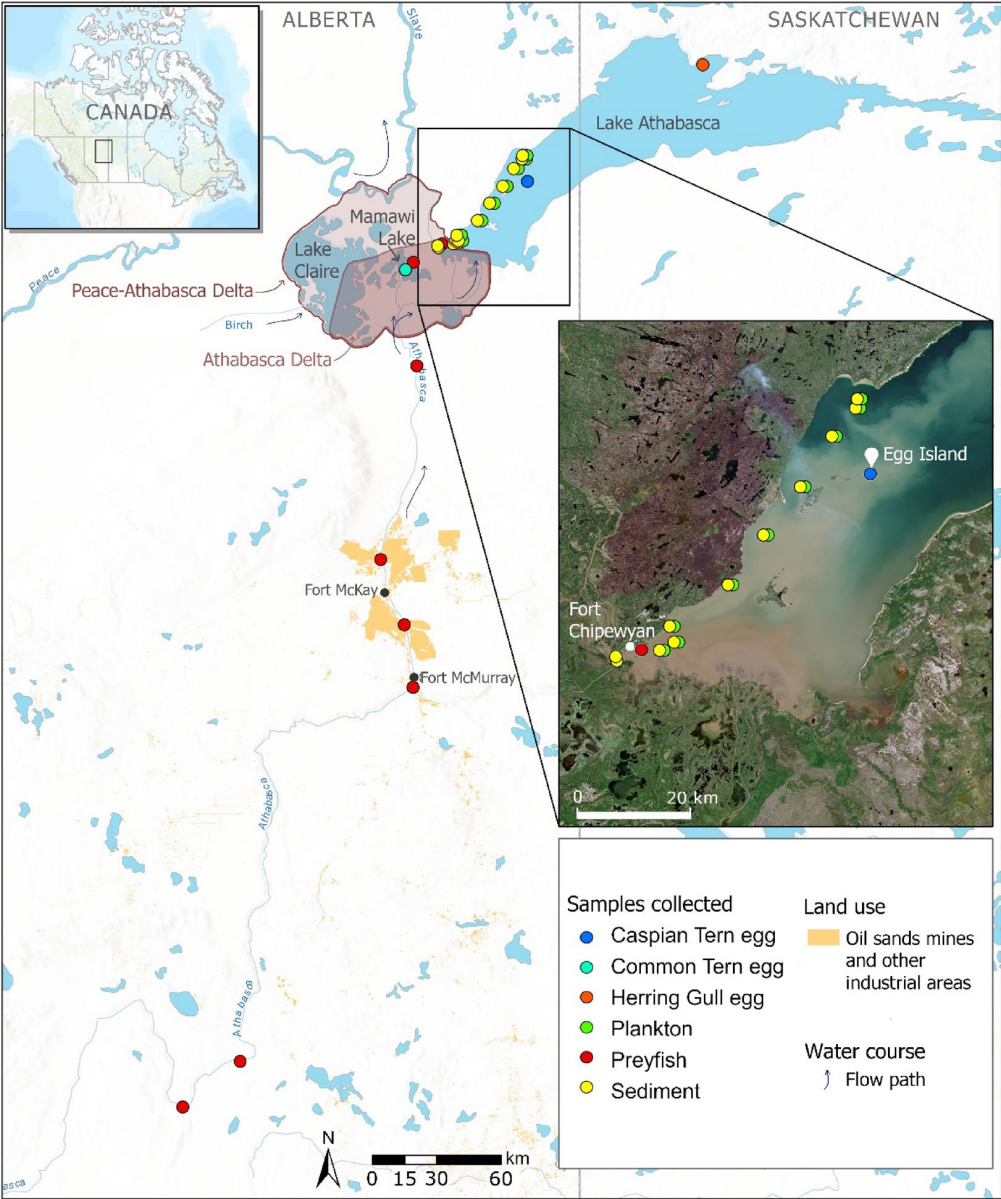
Map of the study area in the provinces of Alberta and Saskatchewan (Canada), including collection locations of sediment, plankton, fish and colonial waterbird eggs. Map generated with ArcGIS Pro 3.2.2 (www.esri.com; mapping credits: Esri, TomTom, Garmin, FAO, NOAA, USGS, EPA, NRCan, Parks Canada, CGIAR, Canadian Community Maps contributors) and edited with GIMP 2.8.22 (www.gimp.org). The inset image of western Lake Athabasca was obtained courtesy of Sentinel-2 data (www.browser.dataspace.copernicus.eu) and taken on June 25, 2023, one month before the survey.

The Peace-Athabasca Delta (PAD) in northern Alberta, Canada, is one of the largest boreal freshwater deltas in the world^13^. This region is of broad ecological and cultural importance to local Indigenous Peoples, and the PAD has been recognized as a World Heritage Site of international ecological significance for its wetland habitats^14^. It is protected within the largest national park in Canada, Wood Buffalo National Park. The PAD receives inflows from three rivers, the Peace, Birch and Athabasca, forming interconnected deltas that cover over 3000 km^2^ (Fig. 1). The Athabasca River, which flows into the Athabasca sector of the PAD (hereafter referred to as the Athabasca Delta) is the main source of water entering western Lake Athabasca^13,15^. It is possible for water and sediment from the Peace River to drain into the Athabasca Delta and Lake Athabasca; however, this occurs only in rare instances (e.g., twice per decade), when the river level is higher than the water level of the lake and delta^16^. The hydrological influence of the Birch River is minor compared to that of the Athabasca River, having on average less than 5% of the Athabasca’s flow^17^. Beginning in the Rocky Mountains, the Athabasca River drains 159,000 km^2^ of largely coniferous or deciduous forest and boreal wetlands, with some agricultural, urban and industrial development occurring within the watershed^18^. Sporadic permafrost occurs in the lower reaches of the Athabasca River Basin and is vulnerable to thaw with climate warming^19^.

Of notable environmental concern in the region is surface mining of oil sands, which has disturbed approximately 1,055 km^2^ of land (as of 2022) within the Athabasca River watershed^20^. Previous studies have shown trace elements and polycyclic aromatic compounds are deposited on the landscape within approximately 30 km of oil sands mining operations, which could subsequently wash into the Athabasca River^21–23^. Sediment cores collected from lakes near the mines indicated higher atmospheric deposition of lead and vanadium during early years of mining operations^24^. Contaminants released in the watershed may be transported by the Athabasca River to downstream receiving environments^23^.

The hydrology of the Athabasca River downstream of oil sands operations is complex and dynamic, with large seasonal and inter-year variability in discharge. Further, there are many flow paths through the Athabasca Delta which have changed over time^13,16,25–27^. A poorly characterized aspect of downstream water transport is the mixing of river and delta waters in western Lake Athabasca. Remote sensing methods to date have focused on the immediate outflow area of Lake Athabasca^16,28^. In contrast, satellite imagery clearly shows large surface water plumes containing sediment extend farther into the lake throughout the year (Supplementary Fig. S1). The mixing zone of Athabasca River waters in Lake Athabasca requires better characterization to understand the connectivity and downstream fate of contaminants from the Athabasca River including to aquatic food webs.

In this study, we investigated the mixing zone of Athabasca River waters in the large, boreal Lake Athabasca. Dissolved fractions of inorganic and organic carbon in surface waters of the Athabasca River are old^29,30^, and soil particles containing old carbon also erode into the river resulting in a high sediment load^31,32^. The contrast of old dissolved and particulate carbon from the river with a younger carbon pool in Lake Athabasca allowed for quantification of the mixing of river and lake waters. Radiocarbon measurements of surface sediment (as the fraction of modern carbon) were used to trace the influx of old particulate carbon from the Athabasca River into western Lake Athabasca. We then examined the fraction of modern carbon in aquatic biota from the lake and used a Bayesian isotope mixing model (MixSIAR)^33^ to quantify contributions of old river carbon to a modern lake food web. We also examined spatial patterns of metals and trace elements in surface sediments of western Lake Athabasca to characterize the spatial redistribution of chemical constituents transported by the Athabasca River. Our findings on hydrological connectivity, material transport and food web uptake showed the Athabasca River exerts a much larger physical and biological influence in western Lake Athabasca than previously acknowledged.

## Results and discussion

### Longitudinal gradients of sediment and plankton characteristics in western Lake Athabasca

**Fig. 2:**
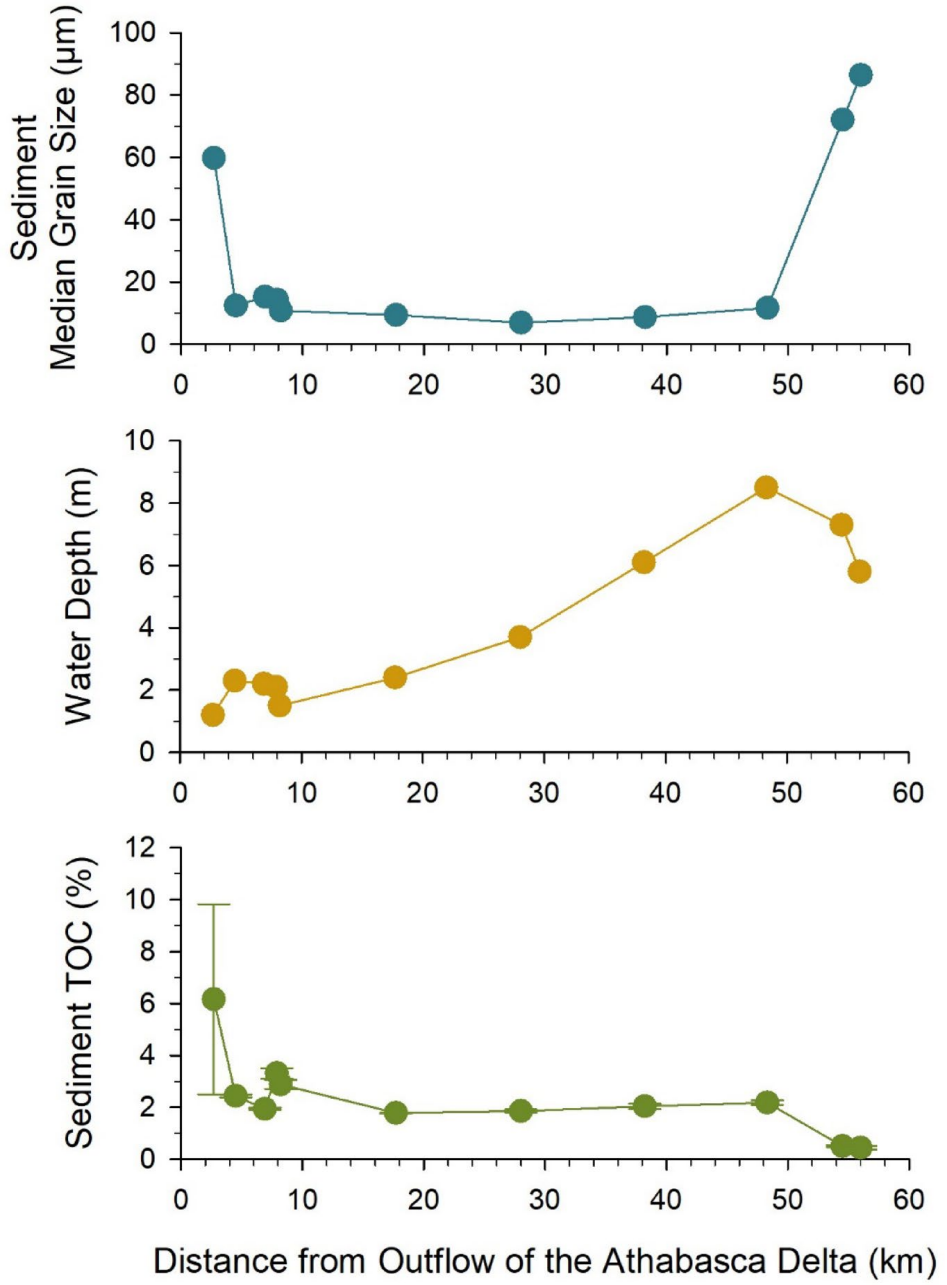
Median grain size and total organic carbon content (TOC) of surface sediment as well as water column depth at sites along a transect in western Lake Athabasca. Data points are individual measurements except TOC, for which the mean and standard deviation of 3 field replicates are presented.

The surface layer of sediment (top ~ 3 cm from grab samples) and water-column plankton (> 200 μm net samples) were collected at 11 sites in western Lake Athabasca along a transect reaching almost 60 km from the outflow of the Athabasca Delta (Fig. 1). The two farthest sites (at kms 54.5–56) were located outside the water column plume of sediment at the time of sampling (see inset image of Fig. 1), while the closer sites were within the plume as indicated by higher turbidity of surface water. Within 20 km of the inflow, water depths were < 3 m (Fig. 2), associated with infilling of river sediment^34^. Deeper waters up to 8 m were sampled at sites farther away.

Spatial patterns of sedimentation were evident for inorganic and organic materials in western Lake Athabasca. The grain size distribution of surface sediments varied along the lake transect (Fig. 2). Sediment was predominately sand (> 50 μm) at the site closest to outflow, reflecting rapid deposition of coarse-sized particles, while silt dominated the sediment (range of median diameter by volume, Dv50 = 7–15 μm) at farther locations within the plume. At the two farthest sites, the surface sediment was again predominately sand. Sediment total organic carbon (TOC) varied in a similar manner with the closest site having higher and more variable organic content (6 ± 4%) associated with the deposition of coarse-sized material, while lower TOC (2 ± 0.5%) occurred in silty sediments (Fig. 2). The lowest sediment TOC (0.5 ± 0.06%) was found in sandy sediments at the two farthest sites, suggesting this area of the basin was less influenced by river inputs of organic matter.

**Fig. 3:**
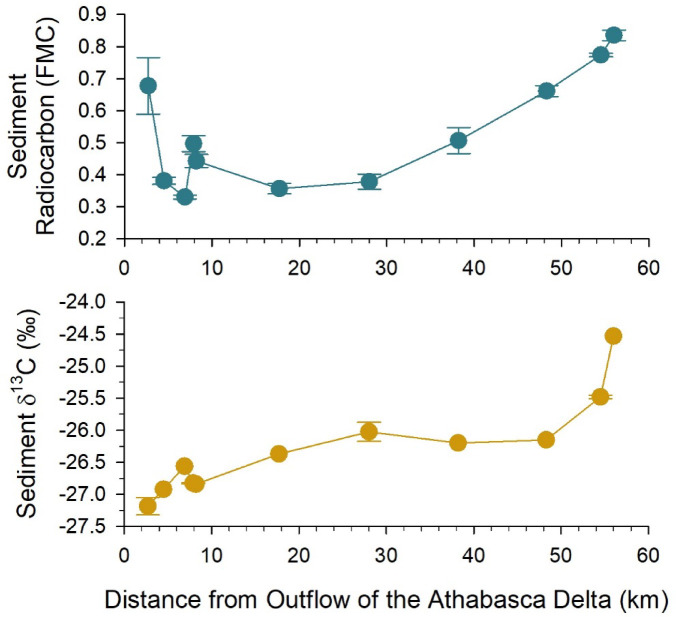
Radiocarbon (fraction modern carbon; FMC) and carbon stable isotope (δ^13^C) values of organic carbon in surface sediment at sites along a transect in western Lake Athabasca. For radiocarbon, data points represent the mean and standard deviation of 3 field replicates per site. For δ^13^C values, data points are individual measurements or the mean and standard deviation of field duplicates per site.

Surface sediments in Lake Athabasca contained old organic carbon (Fig. 3) (F^14^C < 1, referred herein as fraction modern carbon [FMC]), with radiocarbon ages ranging from 1270 (± 15) to 9020 (± 25) ^14^C years before present (BP) (Supplementary Information). The oldest organic carbon was found in surface sediments < 30 km from the Athabasca Delta, and the youngest organic carbon was found in sediments at the two farthest sites. We postulate the spatial pattern of sediment radiocarbon reflects physical mixing of two dominant sources of particulate organic carbon (POC) to the lake: (1) the Athabasca River and Delta, and (2) watershed tributaries entering eastern Lake Athabasca^15^. Although data were not available for the Athabasca River, the radiocarbon activity of POC suspended in waters of the nearby Peace and Slave rivers (range = 0.312–0.367 FMC)^35,36^, which also drain boreal lowland catchments, was comparable to the oldest radiocarbon values of surface sediment in Lake Athabasca (range = 0.330–0.381 FMC; Fig. 3). Likewise, the radiocarbon activity of POC from lowland rivers draining Canadian Shield bedrock in the region (range = 0.748–0.999 FMC)^35,36^ overlapped with the youngest radiocarbon values of surface sediment at the farthest sites on the Lake Athabasca transect (0.773–0.835 FMC; Fig. 3). Variability of sediment radiocarbon activity at sites < 10 km from the Athabasca Delta outflow (FMC = 0.557 ± 215) may reflect turbulent transport and heterogenous deposition of coarse organic carbon from differently aged sources (e.g., modern and old plant material, soil). Overall, the longitudinal pattern of organic radiocarbon in surface sediments showed an influence of inputs from the Athabasca River and Delta for at least 50 km into western Lake Athabasca.

The stable isotope values of organic carbon (δ^13^C) in surface sediment suggested inputs of terrestrial organic matter were important in western Lake Athabasca. At sites located < 50 km from the Athabasca Delta, surface sediment δ^13^C values averaged − 26.6 ± 0.4‰ and were similar to the average signature of terrestrial plant material (~−27‰ for C3 plants)^37,38^ (Fig. 3). At the two farthest sites, organic carbon δ^13^C values in sediment were significantly more positive (average = −25.2 ± 0.5‰; *t*-test, *t* = −4.889, *p* < 0.001, *n* = 15). For reference, POC δ^13^C values of −27.0 to −25.6‰ have been reported for the Peace and Slave rivers, as well as lowland rivers draining Canadian Shield in the region^35,36^. There was a gradual increase in sediment δ^13^C values with distance from the Athabasca Delta, indicating a mixing of river organic carbon with organic carbon in the lake, the latter having more positive values (Fig. 3). One of river sources of organic carbon was likely shoreline soils in the lower reach of the Athabasca River (e.g., McConnell Mud, Prodelta sediment), which are known to contribute to sedimentation in the Athabasca Delta and beyond^32,39^.

**Fig. 4:**
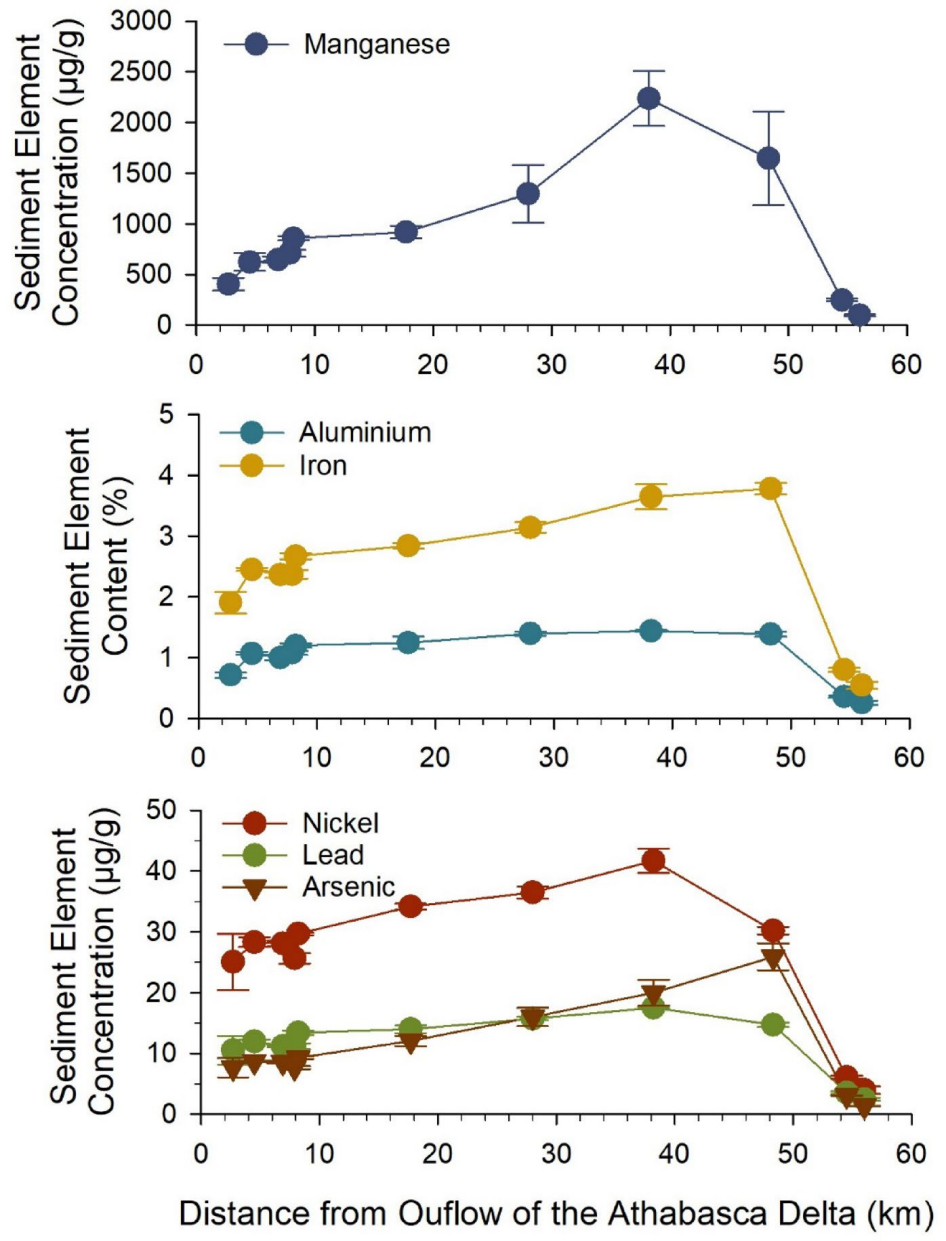
Metal and metalloid concentrations of surface sediments at sites along a transect in western Lake Athabasca. Data points represent the mean and standard deviation of 3 field replicates.

Element concentrations of surface sediment were examined as additional tracers of particle transport from the Athabasca River into western Lake Athabasca. Spatial gradients of element concentrations in surface sediment were related to particle size and/or TOC. A principal component analysis (PCA) showed that particle size was the primary driver of spatial variability for most elements, with higher sediment concentrations associated with lower median particle size (Dv50) (Supplemental Fig. S2). Likewise, concentrations of most elements examined (23 of 25 elements) were negatively correlated with median particle size (Pearson *r* = −0.49 to −0.95, *p* ≤ 0.004, Supplemental Fig. S3, Table S1). This spatial pattern is consistent with the process of sediment focusing in lakes, where fine sediments are resuspended in shallow areas by wave action and laterally transported to deeper low-energy sediments where less disturbance occurs^40,41^. For those elements, concentrations increased with water depth and distance from the Athabasca Delta (Fig. 4). For example, sediment concentrations of manganese, iron and aluminum were 3, 1.6 and 1.3 times higher, respectively, at the farthest sites in the plume (kms 38–48) relative to sites < 10 km from the Athabasca Delta (Fig. 4). Similarly, arsenic, nickel and lead were 1.3–2.7 times higher in deeper, farther sites, coinciding with enrichment of manganese and iron (Fig. 4). Some elements such as calcium, mercury and sulfur were also positively correlated with sediment TOC (Pearson *r* = 0.42–0.83, *p* ≤ 0.015) and declined with distance from the Athabasca Delta (Supplemental Fig. S2, S3, Table S1, S2). The lowest concentrations of every element were found in surface sediments at the two farthest sites (Supplementary Table S2), which were outside the zone influenced by lateral transport of fine particles and organic carbon. Concentrations and enrichment factors in surface sediments are provided for all measured elements in Supplemental Table S2.

The longitudinal distribution of trace elements in surface sediments provides a second line of evidence, in addition to radiocarbon, that the depositional area of Athabasca River outflow extends far into western Lake Athabasca. Previous research has examined sedimentation processes in the PAD and at the outflow of the Athabasca River within 10 km of the delta, focusing on geomorphological change associated with coarse particulates^16,27,34,42^. Our findings, which included farther sites in western Lake Athabasca, indicated that wave action and water currents are transporting fine particles at least 50 km into the lake. Higher concentrations of some elements were found farther away, and therefore, this large zone of influence is relevant for understanding the spatial redistribution of chemical consistuents transported by the Athabasca River.

### Incorporation of old river carbon in aquatic food webs of Lake Athabasca and the PAD

**Fig. 5:**
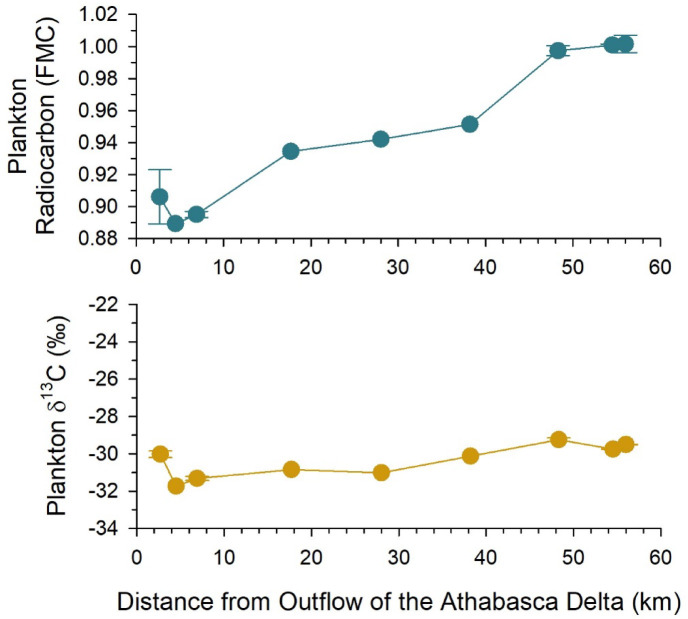
Radiocarbon (fraction modern carbon; FMC) and carbon stable isotope (δ^13^C) content of plankton (> 200 μm) at sites along a transect in western Lake Athabasca. Data points are individual measurements or the mean and standard deviation of field duplicates.

Plankton in the water column of Lake Athabasca contained old organic carbon at sites < 40 km from the Athabasca Delta and modern carbon (FMC ≥ 1) at farther sites (Fig. 5). The lowest radiocarbon ages of plankton, at sites < 10 km from the Athabasca Delta, were estimated at 690 (± 15) to 945 (± 15) ^14^C years BP. The FMC of plankton increased with distance from the delta. The plankton were bulk samples (> 200 μm) composed primarily of present-day zooplankton and filamentous phytoplankton, determined by visual inspection and δ^13^C measurements. The δ^13^C values of plankton averaged − 30.2 ± 0.8% (Fig. 5) and were distinctly lower than organic carbon from surface sediments (*t*-test, *t* = 13.541, *p* < 0.001, *n* = 29; Figs. 3 and 5). Plankton δ^13^C values were consistent with those typically reported for autochthonous pelagic carbon found in lakes^43,44^. Likewise, the δ^13^C of plankton was lower than values for POC in regional rivers (e.g., Peace and Slave rivers, −27.0 to −25.6‰)^35,36^.

We postulate that old radiocarbon signatures of plankton reflect the photosynthetic fixation of dissolved inorganic carbon (DIC) from the Athabasca River by phytoplankton in Lake Athabasca. Surface water DIC of the lower Athabasca River has an average DIC radiocarbon value of 0.872 FMC (range = 0.859–0.895 FMC, *n* = 6)^29^, which is similar to our plankton samples at sites close to the Athabasca Delta (range = 0.889–0.918 FMC, Fig. 5). Further, two macro-algal samples collected at the Lake Athabasca shoreline close to the delta had similarly low radiocarbon values (filamentous green algae and *Chara*, 0.86–0.92 FMC), consistent with autochthonous uptake of old DIC. An increase in the FMC of plankton with distance from the delta indicated a gradient in mixing of river waters with lake waters, the latter likely composed of predominately modern DIC^10,45^. However, other processes could be driving the radiocarbon signature of plankton. Bacterial consumption and respiration of old organic matter in the water column of Lake Athabasca^46^ and subsequent incorporation into the food web could have contributed to the observed patterns^47^. Likewise, plankton sampled near the Athabasca Delta may have originated in the lower Athabasca River or Delta and then were transported into the lake, rather than in situ growth of plankton within the lake. Regardless of the mechanism, the longitudinal variation of plankton radiocarbon showed the important river influence on the food web far into Lake Athabasca.

**Fig. 6:**
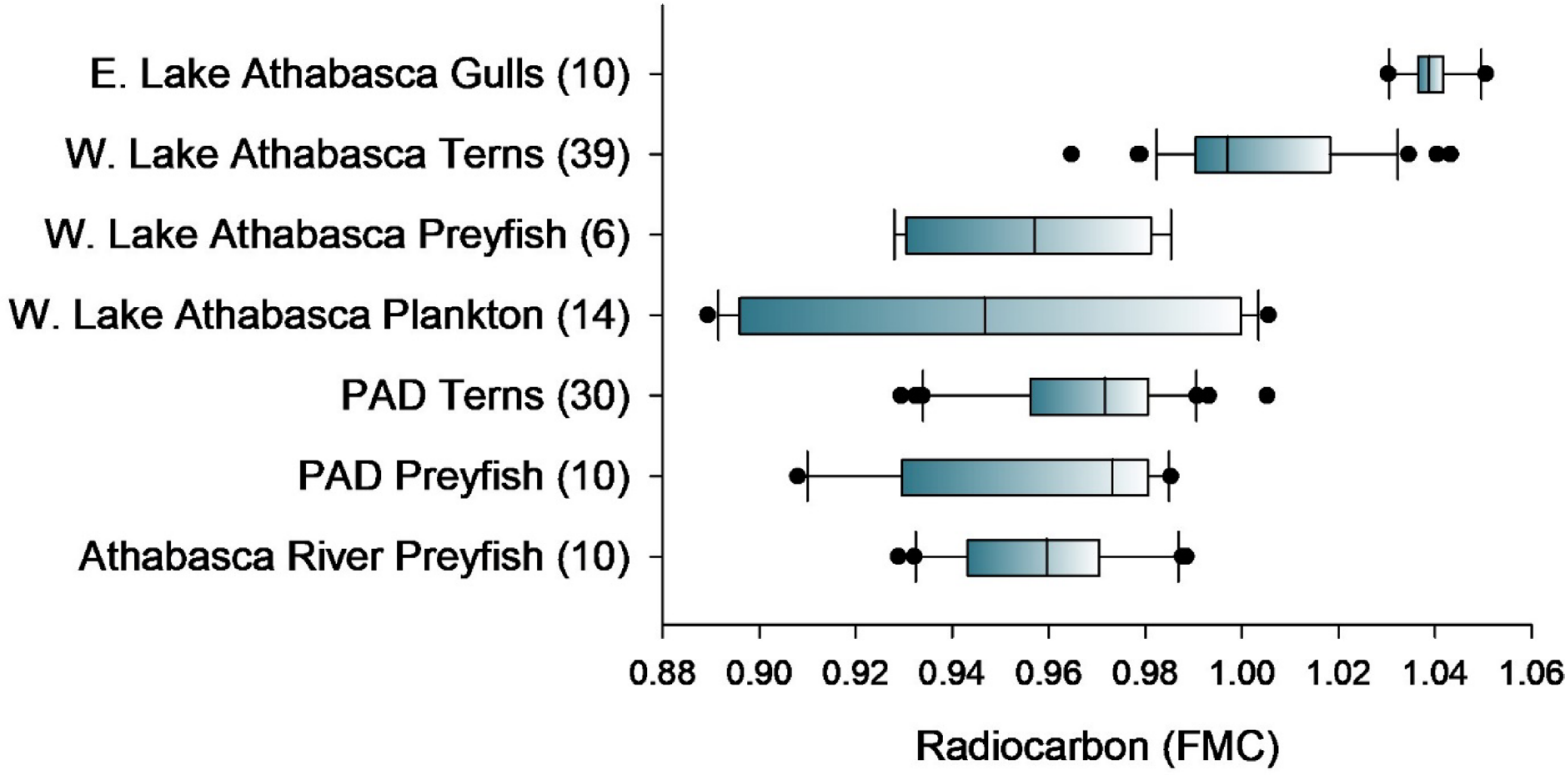
Radiocarbon content (fraction modern carbon, FMC) of plankton, fish and colonial waterbirds from aquatic food webs of the Athabasca River, Peace-Athabasca Delta (PAD), and Lake Athabasca. Note that biota from Lake Athabasca are separated by western (W.) and eastern (E.) basins. Shiner species were analyzed for small-bodied preyfish and eggs were analyzed for Common Tern (PAD), Caspian Tern (Lake Athabasca) and Herring Gull (Lake Athabasca). Sample sizes are in parentheses.

Present-day vertebrates from aquatic food webs in the study area were also analyzed for radiocarbon and the vast majority had old carbon (FMC < 1) incorporated in their tissues (Fig. 6). We focused on small-bodied preyfish (two shiner species) from sites on the Athabasca River, Mamawi Lake in the PAD, and western Lake Athabasca as well as eggs of terns and gulls breeding on Mamawi Lake and Lake Athabasca (see Fig. 1 for site locations). The chemical composition of colonial waterbird eggs, including terns and gulls, reflects the local diet of the female at the time of breeding^48,49^. Thus, eggs are an indicator of dietary exposure to fish-eating waterbirds at the top of food webs. We found that preyfish sampled at all sites in the large river-wetland-lake complex had radiocarbon values < 1 FMC. The frequency of tern eggs with FMC < 1 at breeding colonies in the PAD and western Lake Athabasca was 97% and 54%, respectively. In comparison, gull eggs from a site in eastern Lake Athabasca (Fig. 1), farther away from the influence of the Athabasca River, were all composed of modern carbon (of atmospheric origin), as expected for biota in the pelagic food web of large lakes^10,50^.

**Fig. 7:**
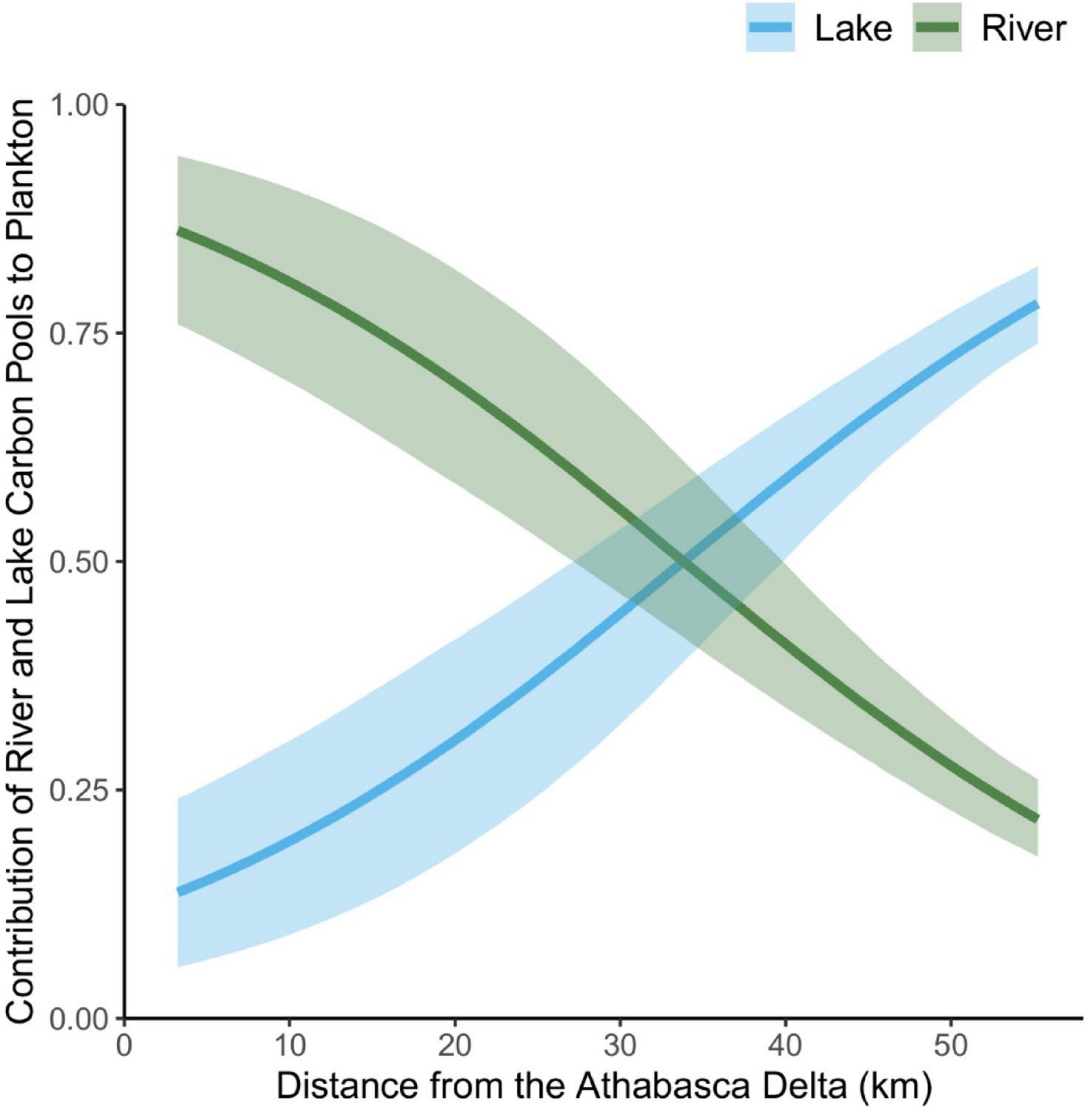
MixSIAR model estimates (median and 95% credible interval) of the relative contributions of old river carbon and modern lake carbon to plankton biomass in Lake Athabasca with increasing distance from the Athabasca Delta.

A two-source Bayesian mixing model (MixSIAR)^33^ was used to estimate the contributions of old carbon from the Athabasca River and modern carbon from Lake Athabasca in the biomass of plankton, prey fish, and tern eggs. The radiocarbon signature of the river end-member was estimated using surface water DIC of the Athabasca River^29^, and the signature for the lake end-member was estimated using gull eggs from a far-away breeding site in eastern Lake Athabasca (Fig. 1). Gull eggs were used as a proxy due to the absence of radiocarbon data for water DIC of Lake Athabasca and assuming this top predator reflected the radiocarbon signature of the lake food web outside of the river’s influence. Model estimates (median and 95% credible interval) showed the proportion of old river carbon in plankton biomass was > 75% near the Athabasca Delta and declined with distance from delta to ~ 25% at 50 km (Fig. 7). Two other food web components, prey fish and tern eggs, showed comparable results. Prey fish from a shoreline site < 10 km from the Athabasca Delta were on average composed of 49% (95% credible interval = 35–67%) old river carbon. The local diet of terns breeding in western Lake Athabasca (at a site ~ 40 km from the delta) was on average composed of 21% (16–26%) old river carbon. These estimates indicated the Athabasca River and Delta are a substantial source of old carbon subsidies to the modern food web of a large lake.

### Relevance for carbon cycling in large lakes

This study presents a novel example of important subsidies of old carbon to sediment and the food web of a large boreal lake. While utilization of old carbon has been demonstrated for rivers and smaller lakes^5,9,51^, our ~ 60 km transect and food web measurements tracked the movement of river carbon far into Lake Athabasca. To our knowledge, this study is the first to demonstrate significant incorporation of old carbon to the food web of such a large lake (covering 7,850 km^2^). Satellite imagery of the suspended sediment plume in western Lake Athabasca suggests that an area of over 1500 km^2^ may be influenced by river inputs, though more detailed satellite and field measurements are needed (Supplementary Fig. S1, S4). The findings underscore the high connectivity of a river-delta-lake complex that is enhanced by relatively shallow depths in western Lake Athabasca and a large volume of water and sediment flushing in from the Athabasca River.

A second novel aspect of this study is the finding that old carbon substantially supported the biomass of top predators, specifically fish-eating terns. Old carbon is thought to enter food webs primarily via the microbial loop, where dissolved and particulate organic matter is metabolized by microbes which are then consumed by protists and invertebrates^5,9^. As such, the research on food web incorporation of old carbon has largely focused on primary consumer organisms, particularly zooplankton and benthic invertebrates^2,5,9,51^. The subsidies of old carbon to the local diet of breeding terns in the delta and lake (Fig. 6) suggested high bioavailability of the allochthonous carbon and a broad contribution to the base of the food web supporting top predators. A possible explanation is that large river loads of old DIC are highly bioavailable and more efficiently taken up by phytoplankton compared to old carbon uptake via the microbial loop.

Further research is needed to refine our model estimates of old carbon contributions to the food web through radiocarbon measurements of different carbon source pools (e.g., lake and river DIC, as well as dissolved and particulate organic carbon). The pathways leading to food web uptake of old carbon also need to be characterized: the incorporation of old DIC during primary production or bacterial metabolism of old organic matter^5^. Additionally, research should focus on identifying the dominant watershed sources of old carbon transported to Lake Athabasca, including groundwater, weathering of carbonates, thawing of permafrost, peatlands, and soil organic matter^19,29,46^. These research avenues will advance our understanding of carbon cycling in large boreal ecosystems including to support estimates of regional carbon budgets^12,52^.

### Implications for environmental management of Lake Athabasca

The findings on the importance of western Lake Athabasca as a receiving environment and depositional area of the Athabasca River underscore the need for greater environmental study of this important waterbody. Four large environmental programs have been implemented in the Athabasca River Basin since the 1990s. The Northern River Basins Study, from 1991 to 1994, was a large-scale investigation of pollution in the Athabasca River Basin that included measurement of water and sediment chemistry in western Lake Athabasca^53,54^. Since 2000, surface water quality has not been routinely measured in western Lake Athabasca by the provincial government^55^. Subsequent provincial and federal government initiatives, namely the Regional Aquatics Monitoring Program (1997–2011), the Joint Oil Sands Monitoring program (2012–2015), and the Oil Sands Monitoring program (2017-present) have focused on evaluating environmental impacts of the oil sands industry in the river basin^56^. None of those programs included a long-term site for water or sediment quality in western Lake Athabasca. To our knowledge, there are 3 published studies with sediment chemistry data for western Lake Athabasca, and most samples were collected at locations < 10 km from the Athabasca Delta^54,57,58^. In contrast, over 200 studies have reported on water and sediment quality upstream in the river itself^59^; clearly there is a paucity of environmental data for a lake of such size and importance.

The woeful lack of water and sediment measurements for Lake Athabasca may at least partly stem from a long-standing assumption, based on old data, that waters of the Athabasca River typically only reach about 10–15 km offshore from the delta^42,54,60^. Since the outflow of Lake Athabasca is very close to the inflow of the river, it has largely been assumed that the river waters quickly drain westward and only enter the lake more appreciably under high flows or high winds^60^. The radiocarbon data tell a different story regarding the importance of river fluxes to the lake. Satellite imagery also shows that sediment plumes regularly disperse far into the lake across months of the open water season (Supplementary Fig. S1).

Looking to the future, more comprehensive research is needed to characterize the carbon sources and uptake pathways that lead to biological uptake of old carbon in this boreal river-wetland-lake complex. Radiocarbon was a powerful tracer that demonstrated connectivity within the Athabasca River Basin and the importance of the mixing zone in western Lake Athabasca, which is also the receiving waters for a suite of chemical stressors from agriculture, pulp mills, municipal wastewater, and oil sands operations within the basin^54,59^. This relevance extends to future consideration of treated effluent release into the Athabasca River as a strategy to manage the 1.4 trillion L of toxic tailings pond water that has accumulated from oil sands operations^61^. Globally, radiocarbon has the potential to effectively track the fate of large-scale material transport of major rivers to downstream receiving environments.

## Methods

### Study area

The study area encompassed the lower reaches of the Athabasca River Basin, including the Athabasca River (upstream and downstream of oil sands operations), the Athabasca sector of the Peace-Athabasca Delta (herein referred to as the Athabasca Delta) and Lake Athabasca (Fig. 1). This region falls within the boreal plains ecozone of northern Alberta (latitude ~ 55–59 °N). The Athabasca River originates in the Rocky Mountains of British Columbia and drains a watershed of 159,000 km^2^ of largely coniferous and deciduous forest (71%) and wetlands with some agricultural, urban and industrial development (< 10%) (land use data for Alberta only)^18^. The mean flow of the Athabasca River is lowest in winter (165 ± 45 m^3^/s, December to February, 2000–2023) and highest in summer (1141 ± 454 m^3^/s, June to August, 2000–2023), though there is large inter-year variability in flow^62^. As the Athabasca River passes through the delta, the water flows into Lake Athabasca via multiple channels with complex hydrological connections (including to perched wetlands) that vary with water level^63,64^. Lake Athabasca receives the inflow of the river and delta at the southwest end of the lake, close to the lake outflow that drains to the Slave River downstream. The Athabasca River is the main source of surface water to the lake (and is an important driver of the lake water level), though other smaller rivers and tributaries also contribute via drainage into eastern Lake Athabasca^13,15,63^ within the province of Saskatchewan (Fig. 1). The bathymetry of Lake Athabasca remains poorly characterized, and spot measurements from the only available nautical chart (Canadian Hydrographic Service, chart CHS6310) indicate the western section is shallow with depths < 10 m, while the eastern basin is relatively deep (typically > 50 m, maximum depth 124 m). There is no agricultural or industrial development on the shores of western Lake Athabasca and only one community, Fort Chipewyan (population approximately 800), on the southwest shore near the outflow.

### Sampling

Surface sediment and plankton were surveyed by boat along a ~ 60 km transect in western Lake Athabasca on July 24–27, 2023. A total of 11 sites were sampled and the mouth of the Embarras River was used as the point of reference to estimate distance from the Athabasca Delta (river inflow to the lake). The two farthest sites were located outside the sediment plume (at the time of sampling) as indicated by high water clarity. At each site, surface sediments were sampled in triplicate with three separate Ekman grabs. Approximately 50–100 g wet weight of sediment was scooped per sample from the top 3 cm of the grab and placed in a whirlpak bag. Single or duplicate plankton samples were collected from each site by horizontal tows of a large plankton net (mesh size 200 μm, 1 m diameter opening) near the water surface by boat. Plankton samples were placed in trace-metal clean 250 mL HDPE jars. Visual inspection of net contents suggested samples were composed primarily of zooplankton and macro-filamentous phytoplankton. Plankton were not collected from two shallow sites (~ 2 m depth) near the Athabasca Delta due to macrophyte debris found in the net and water column. Two macro-algae samples (filamentous green algae and the macrophyte *Chara* spp.) were collected by hand from a shoreline area of western Lake Athabasca at the community of Fort Chipewyan. Sediment, plankton and macro-algae samples were frozen on the day of collection.

Samples of vertebrate animals, representative of local aquatic food webs, were obtained from archived collections of the Oil Sands Monitoring program. Whole-body samples of two preyfish species, Emerald Shiner (*Notropis atherinoides*) and Spottail Shiner (*Notropis hudsonius*), were collected in shallow, shoreline areas between 2017 and 2019 through community-based and Environment Canada monitoring. Preyfish were obtained from eight sites including on the Athabasca River (upstream and downstream of Oil Sands operations), Mamawi Lake (in the PAD) and western Lake Athabasca. A total of 36 preyfish samples were either individual fish or pools of 2–5 fish. Eggs of fish-eating terns and gulls were collected in June over multiple years from breeding sites on Mamawi Lake (Common Tern, *Sterna hirundo*, *n* = 30, 2015–2019), Egg Island on western Lake Athabasca (Caspian Tern, *Hydroprogne caspia*, *n* = 39, 2018–2022), and an unnamed island on eastern Lake Athabasca (Herring Gull, *Larus argentatus*, *n* = 10, 2014) (see Fig. 1)^65,66^. The chemical composition of the eggs reflects the local diet of the female at the time of breeding^48,49^.

### Laboratory analyses

Sediment, plankton, macro-algae and vertebrate samples were homogenized and freeze-dried prior to chemical analysis. Dried sediment, macro-algae, and plankton were homogenized by acid-washed mortar and pestle, while preyfish and egg contents (shell excluded) were homogenized by ball-milling prior to drying.

A total of 164 samples were analyzed for radiocarbon by accelerator mass spectrometry at the André E. Lalonde AMS Laboratory at the University of Ottawa (Ottawa, Canada). Dried and homogenized samples were pre-treated with acid to removed carbonates (HCl, 1 N, 80 °C, 30 min) followed by triple rinsing with Milli-Q water and freeze-drying^67^. Pre-treated, freeze-dried samples were then converted to carbon dioxide with a ThermoFlash elemental analyzer and converted to graphite using a semi-automated process outlined in Cran et al.^67^. Radiocarbon analysis was performed on an Ionplus AG MICADAS (Mini Carbon Dating System). The fraction modern (F^14^C) was calculated according to Reimer et al.^68^ as the ratio of the sample ^14^C/^12^C to that of primary standard oxalic acid (OX-II). Both ^14^C/^12^C ratios were background-corrected and fractionation-corrected using the online AMS measured ^13^C/^12^C ratio and normalized to δ^13^C (Vienna PeeDee Belemnite standard). Conventional radiocarbon ages (^14^C yrs. BP) were calculated as −8033ln(F^14^C) and reported in ^14^C yr BP (where BP = AD 1950) according to Stuiver and Polach^69^.

Particle size analysis of surface sediment was performed at SGS Environmental Services (Lakefield, ON, Canada) on a Malvern Panalytical Mastersizer. This instrument uses laser diffraction to measure particle size and the size distribution on a volume basis. Sediment used for this analysis was aliquoted prior to drying and homogenization. One sediment sample per site was measured for particle size distribution, and the median diameter of particles on a volume basis, Dv50, is reported.

Dried and homogenized surface sediment (*n* = 33) was analyzed for total organic carbon (TOC) and concentrations of 37 elements at a commercial laboratory (Bureau Veritas, Vancouver, Canada). A sample of 0.5 g dry weight was digested in a modified aqua regia solution (1:1:1 HNO_3_:HCl: H_2_O) prior to detection by inductively coupled plasma mass spectrometry. The acid digestion used by the commercial laboratory targeted non-mineralized to weakly mineralized elements, and therefore reported concentrations should be considered as labile rather than total concentrations. Sediment TOC was analyzed by combustion on a Leco carbon analyzer. Quality assurance and quality control measures included analytical duplicates (*n* = 2), blanks (*n* = 2), an internal standard (*n* = 2), and a certified reference material (OREAS 262, *n* = 2). For the 25 reported elements (with sample results consistently above analytical detection), blank results were below or at analytical detection limits (96% and 4% of elements, respectively). The relative percent difference between duplicate element concentrations was 3 ± 6%. Recoveries of elements from an internal standard and a certified reference material averaged 95 ± 8%.

Dried and homogenized surface sediment (*n* = 15) and plankton (*n* = 14) were analyzed for carbon stable isotope ratios at the Ján Veizer Laboratory at the University of Ottawa (Ottawa, Canada). To remove carbonates, sediment samples were pre-treated with 10% HCl for two days, rinsed with distilled deionized water, and dried in an oven at 50 °C. Samples and standards were weighed in tin capsules and combusted in a Vario EL Cube elemental analyzer interfaced to a Thermo Delta Advantage isotope ratio mass spectrometer. The δ^13^C results were reported as the per mil (‰) deviation from the Vienna PeeDee Belemnite standard. Duplicates and a standard (glutamic acid) were analyzed every 10 samples and were within 0.2‰.

### Data analysis

Radiocarbon results are reported as the fraction modern carbon (F^14^C, referred herein as FMC), which is the F^14^C in the environmental sample relative to the theoretical F^14^C of the atmosphere without human disturbance (i.e. bomb testing)^68^. This radiocarbon measurement is not dependent on the year of analysis and is recommended for post-bomb applications (i.e. after 1950)^70^. Radiocarbon data are expressed as FMC in the paper, although results for the samples expressed as δ^14^C and Δ^14^C are also available in the Supplemental Information.

Elements with concentrations largely below or close to analytical detection were not included in the data analysis. A total of 25 out of 37 measured elements were examined for longitudinal trends along the sediment transect in western Lake Athabasca. A principal component analysis was performed in R programming environment (prcomp function, data centered and scaled) to evaluate gradients of raw element concentrations in relation to particle size and TOC of surface sediments. Pearson correlations were tested in R (cor function) between element concentrations and particle size or TOC to quantify the influence of those two sediment characteristics.

Using MixSIAR^33^ in R programing environment, Bayesian mixing models were run to estimate the contributions of carbon pools (as FMC) from the Athabasca River versus Lake Athabasca to the biomass of Caspian Tern eggs (*n* = 39), preyfish (*n* = 6) and water column plankton (*n* = 14) from Lake Athabasca. The end-members for the models were derived from published data for DIC in the Athabasca River (mean = 0.872, sd = 0.023, *n* = 6)^29^ and from measurements of Herring Gull eggs collected from eastern Lake Athabasca (mean = 1.039, sd = 0.0055, *n* = 10) (see Fig. 1). We assumed no fractionation of radiocarbon activity during biological uptake or trophic transfer^10^. Models were run using the “long” Monte-Carlo Markov Chain preset provided by MixSIAR (chain length = 300,000; burn-in = 200,000; thin = 100; no. chains = 3). While no fixed or random effects were explicitly included in either model, each model was run separately for a single sample type (Caspian Terns, preyfish or plankton). Within the plankton model, distance from the Athabasca Delta was included as a continuous effect to capture the spatial gradient in mixing of carbon sources. Convergence of both models was assessed using Gelman-Rubin and Geweke diagnostic tests; all Gelman-Rubin values were < 1.01, and < 5% of Geweke test values fell outside ± 1.96 in each chain.

## Supplementary Information

Below is the link to the electronic supplementary material.

Supplementary Material 1

Supplementary Material 2

## Data Availability

Radiocarbon data used in this publication are available in the Supplementary Information.
