# Supplementary material for: Radiocarbon evidence of river transport and food web uptake of old carbon in Lake Athabasca, Canada
Source: Sci Rep. 2025 Aug 26;15:31455. doi: 10.1038/s41598-025-15926-5 (PMC12381246; doi:10.1038/s41598-025-15926-5)

Supplemental Information for:

Radiocarbon evidence of river transport and food web uptake of old carbon in Lake Athabasca, Canada

John Chételat[1]*, Craig Hebert[1], Christine McClelland[1], Sarah Greenwood[1]

[1] Environment and Climate Change Canada, National Wildlife Research Centre, Ottawa, Ontario, Canada

*Corresponding author, e-mail: john.chetelat@ec.gc.ca

Contents

Figure S1. Satellite images of western Lake Athabasca showing the sediment plume from May to October 2022. 3

Figure S2. Principal component analysis of element concentrations, total organic carbon (TOT.C) and particle size (Dv50) in surface sediments of western Lake Athabasca. 4

Figure S3. Matrix of Pearson correlations between element concentrations, total organic carbon (TOT.C) and particle size (Dv50) in surface sediments of western Lake Athabasca. 5

Table S1. Pearson correlation coefficients and probability values for associations between element concentrations, total organic carbon (TOT.C) and particle size (Dv50) in surface sediments of western Lake Athabasca. 6

Table S2. Total organic carbon (TOC) and element concentrations of surface sediment along a ~60 km transect in western Lake Athabasca. 7

Figure S4. Satellite image of western Lake Athabasca showing the sediment plume on June 15, 2020. 8

Figure S1. Satellite images of western Lake Athabasca showing the sediment plume from May to October 2022. The mean annual flow of the Athabasca River in 2022 (592 m^3/s) was close to the average annual river flow over the previous 15 years (608 m^3/s). The surface area of western Lake Athabasca shown here covers ~1750 km^2. Sentinel-2 satellite images were obtained from Copernicus (https://browser.dataspace.copernicus.eu/).

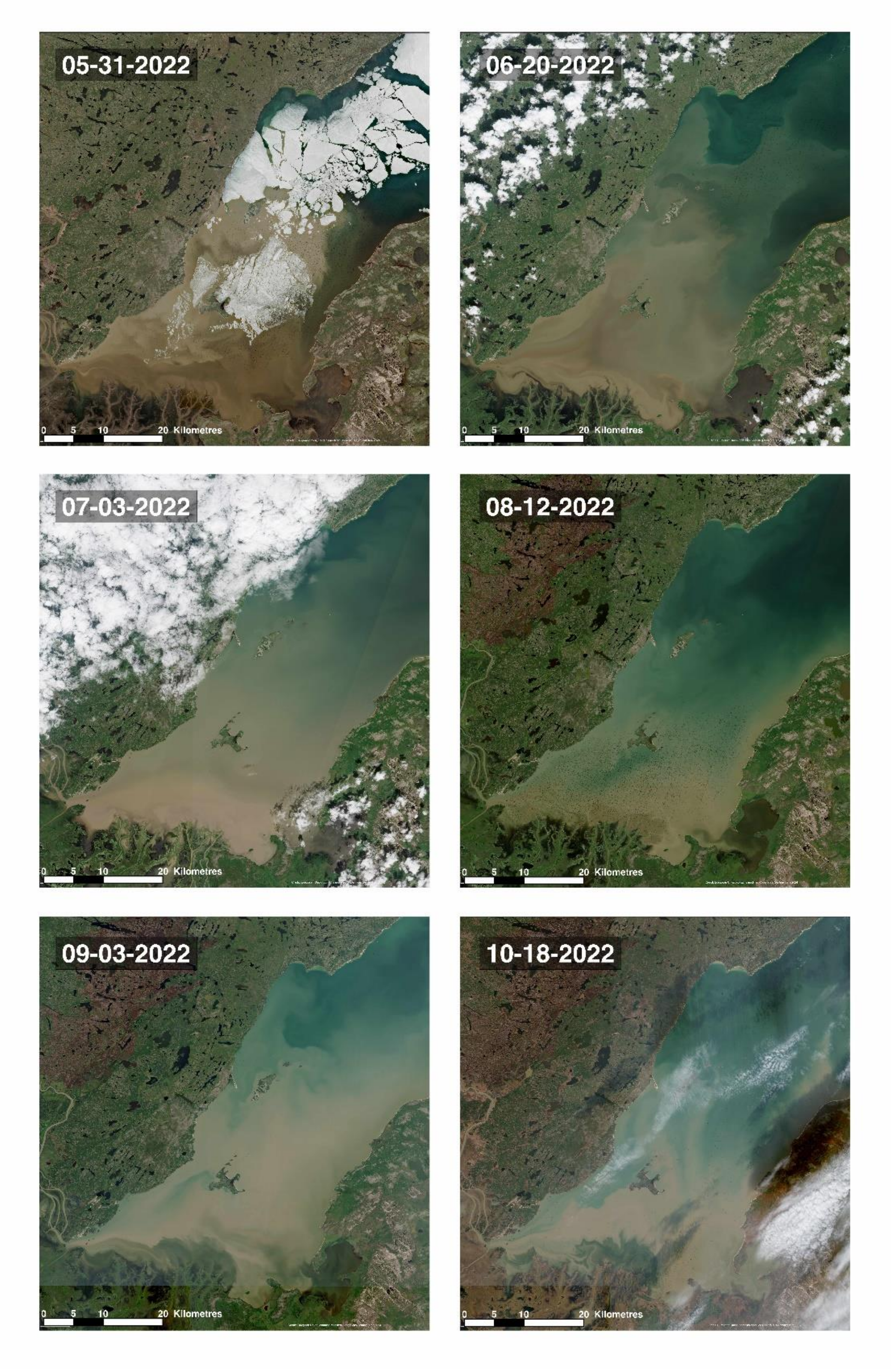

Figure S2. Principal component analysis of element concentrations, total organic carbon (TOT.C) and particle size (Dv50) in surface sediments of western Lake Athabasca. Observations (n = 33) consist of triplicate samples at 11 sites. See Table S1 for concentration units.

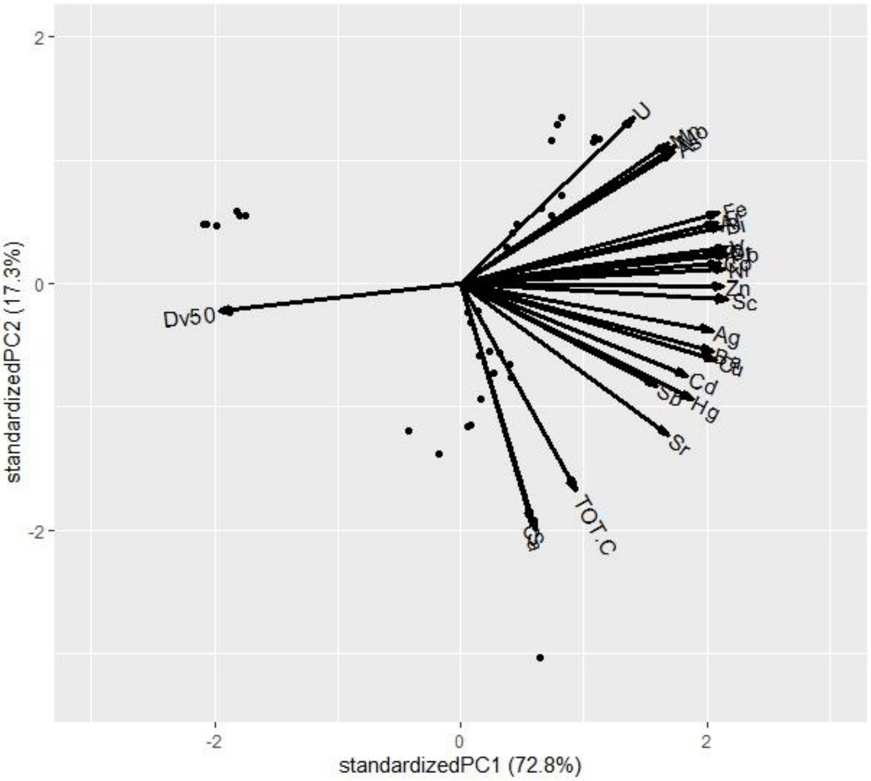

Figure S3. Matrix of Pearson correlations between element concentrations, total organic carbon (TOT.C) and particle size (Dv50) in surface sediments of western Lake Athabasca. Observations (n = 33) consist of triplicate samples at 11 sites. See Table S2 for concentration units.

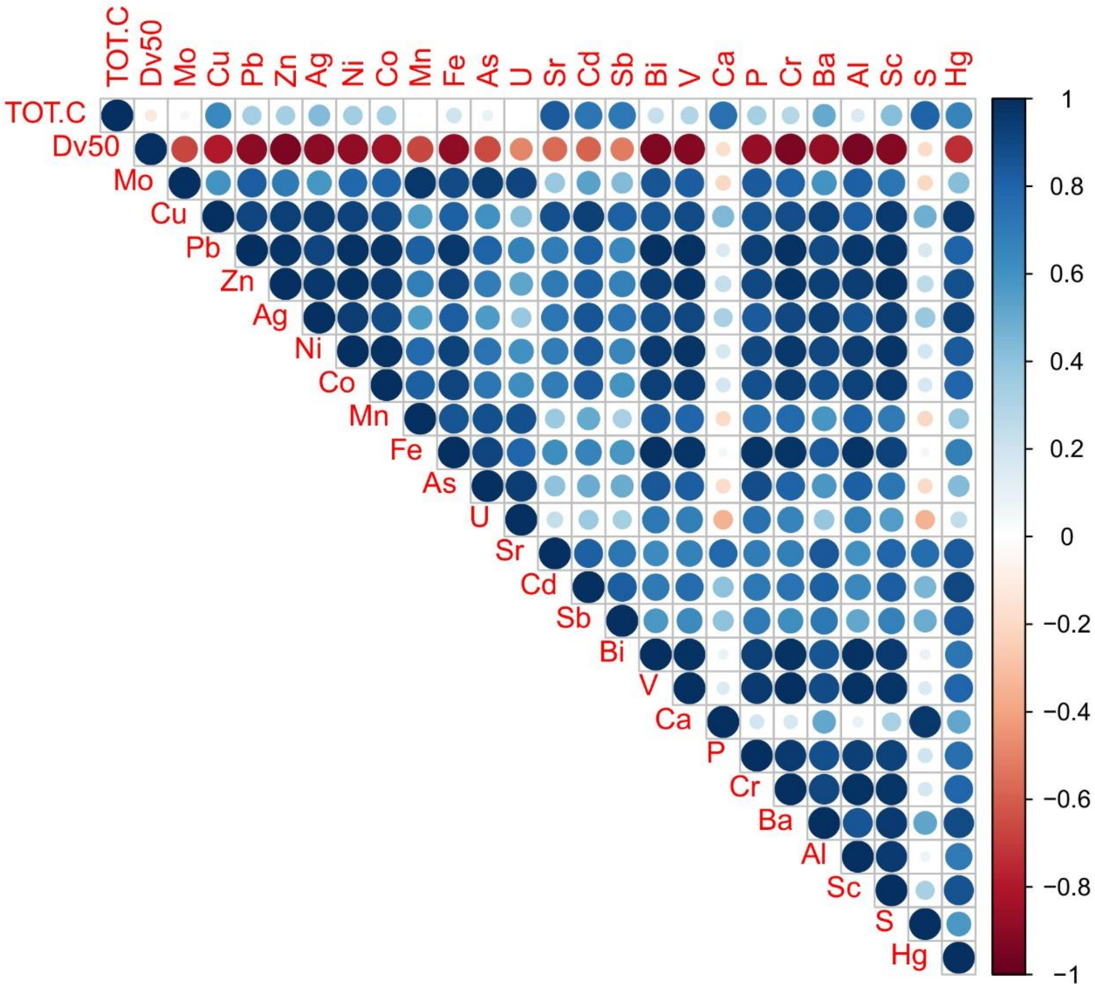

Table S1. Pearson correlation coefficients and probability values for associations between element concentrations, total organic carbon (TOT.C) and particle size (Dv50) in surface sediments of western Lake Athabasca. Observations (n = 33) consist of triplicate samples at 11 sites. Statistically significant correlations are highlighted in bold.

	Pearson coefficients		P-values	
	TOT.C	Dv50	TOT.C	Dv50
TOT.C	1	-0.12		0.5235
Dv50	-0.12	1	0.524	
Mo	0.05	**-0.67**	0.780	**<0.001**
Cu	**0.65**	**-0.8**	**<0.001**	**<0.001**
Pb	0.34	**-0.89**	0.056	**<0.001**
Zn	0.34	**-0.93**	0.056	**<0.001**
Ag	**0.44**	**-0.89**	**0.011**	**<0.001**
Ni	0.34	**-0.89**	0.051	**<0.001**
Co	0.34	**-0.85**	0.053	**<0.001**
Mn	0.01	**-0.66**	0.937	**<0.001**
Fe	0.21	**-0.89**	0.249	**<0.001**
As	0.09	**-0.66**	0.608	**<0.001**
U	-0.01	**-0.49**	0.973	**0.004**
Sr	**0.83**	**-0.57**	**<0.001**	**0.001**
Cd	**0.73**	**-0.58**	**<0.001**	**0.0004**
Sb	**0.71**	**-0.51**	**<0.001**	**0.002**
Bi	0.23	**-0.92**	0.207	**<0.001**
V	0.29	**-0.92**	0.100	**<0.001**
Ca	**0.75**	-0.18	**<0.001**	0.325
P	0.34	**-0.86**	0.057	**<0.001**
Cr	0.28	**-0.94**	0.113	**<0.001**
Ba	**0.50**	**-0.87**	**0.003**	**<0.001**
Al	0.15	**-0.95**	0.394	**<0.001**
Sc	**0.42**	**-0.91**	**0.015**	**<0.001**
S	**0.80**	-0.18	**<0.001**	0.315
Hg	**0.67**	**-0.74**	**<0.001**	**<0.001**

Table S2. Total organic carbon (TOC) and element concentrations of surface sediment along a ~60 km transect in western Lake Athabasca. Mean concentrations are compared between sites within the water column plume of sediment and the two farthest sites located beyond the plume. Enrichment factors associated with particle focusing at deeper offshore sites (within the plume) are also presented (green highlight < 1, blue highlight >1).

Element	Units	Mean concentration (± SD)		Particle-focusing enrichment in plume[c]
		In plume[a]	Beyond plume[b]	
TOC	%	2.73 ± 1.68	0.47 ± 0.06	0.6
Ag	PPB	155.70 ± 21.74	27.17 ± 5.49	1.0
Al	%	1.17 ± 0.23	0.31 ± 0.06	1.4
As	PPM	12.9 ± 6.30	2.25 ± 0.97	2.7
Ba	PPM	196.66 ± 17.64	30.12 ± 6.74	1.0
Bi	PPM	0.22 ± 0.05	0.05 ± 0.01	1.5
Ca	%	1.22 ± 0.86	0.10 ± 0.02	0.2
Cd	PPM	0.49 ± 0.14	0.04 ± 0.01	1.1
Co	PPM	12.55 ± 2.57	2.18 ± 0.62	1.4
Cr	PPM	21.34 ± 3.08	5.75 ± 1.03	1.3
Cu	PPM	23.34 ± 3.30	3.88 ± 0.85	1.0
Fe	%	2.80 ± 0.60	0.68 ± 0.15	1.6
Hg	PPB	66.41 ± 10.23	11.33 ± 4.13	0.9
Mn	PPM	1038 ± 589	173.83 ± 83.66	3.0
Mo	PPM	1.16 ± 0.56	0.27 ± 0.10	2.7
Ni	PPM	31.06 ± 5.44	5.03 ± 1.24	1.3
P	%	0.08 ± 0.01	0.02 ± 0.003	1.4
Pb	PPM	13.38 ± 2.38	3.03 ± 0.63	1.4
S	%	0.09 ± 0.05	0.02 ± 0	0.3
Sb	PPM	0.20 ± 0.05	0.07 ± 0.01	1.0
Sc	PPM	4.53 ± 0.52	0.93 ± 0.16	1.2
Se	PPM	0.86 ± 0.17	0.18 ± 0.04	1.0
Sr	PPM	62.63 ± 12.87	16.18 ± 2.35	0.9
U	PPM	1.79 ± 0.94	0.85 ± 0.18	3.0
V	PPM	32.19 ± 4.91	8.83 ± 1.72	1.3
Zn	PPM	92.89 ± 12.43	15.2 ± 3.50	1.2

[a] Mean element concentration (± SD) for 9 sites within the water column plume of sediment (< 50 km distance from the Athabasca Delta).

[b] Mean element concentration (± SD) for 2 sites located outside the water column plume of sediment (> 50 km distance from the Athabasca Delta).

[C] Ratio of the mean element concentration at shallow sites < 10 km from Athabasca Delta (n = 5 sites) with the mean element concentration at farther deeper sites (40-50 km) still within the water column plume (n = 2 sites). This ratio indicates whether the element concentration was greater at farther sites due to the lateral transport of fine particles.

Figure S4. Satellite image of western Lake Athabasca showing the sediment plume on June 15, 2020. The mean flow of the Athabasca River in 2020 was high (944 m^3/s) compared to the average annual river flow over the previous 15 years (608 m^3/s). The surface area of the sediment plume within the blue polygon covers ~1900 km^2. The Sentinel-2 satellite image was obtained from Copernicus (https://browser.dataspace.copernicus.eu/).

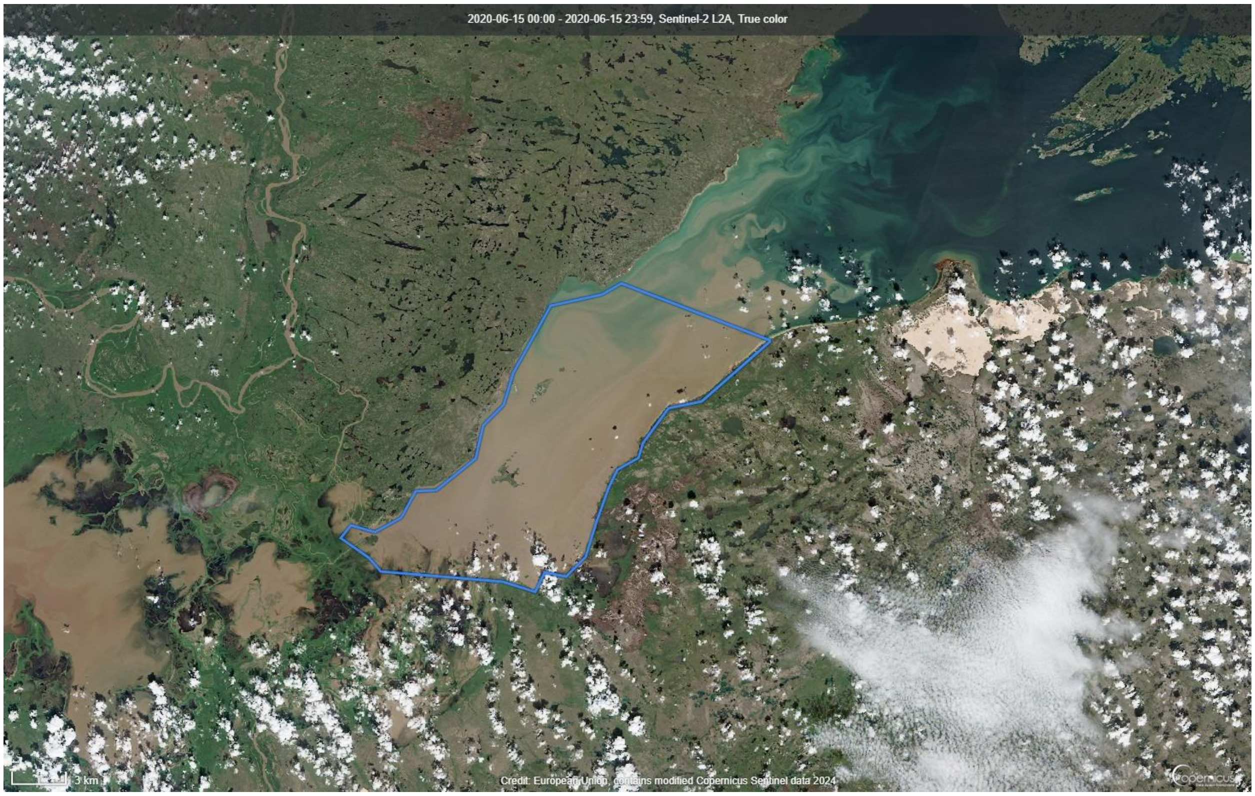

Supplement: Supplementary file 2 — Supplementary Material 2 [file 41598_2025_15926_MOESM2_ESM.pdf]
